# Cascaded Feature Fusion Grasping Network for Real-Time Robotic Systems

**DOI:** 10.3390/s24247958

**Published:** 2024-12-13

**Authors:** Hao Li, Lixin Zheng

**Affiliations:** 1 College of Information Science and Engineering, Huaqiao University, Xiamen 361021, China; lihao@stu.hqu.edu.cn; 2 College of Engineering, Huaqiao University, Quanzhou 362021, China

**Keywords:** RGB-D, robotic grasping, convolutional neural network, grasping pose prediction

## Abstract

Grasping objects of irregular shapes and various sizes remains a key challenge in the field of robotic grasping. This paper proposes a novel RGB-D data-based grasping pose prediction network, termed Cascaded Feature Fusion Grasping Network (CFFGN), designed for high-efficiency, lightweight, and rapid grasping pose estimation. The network employs innovative structural designs, including depth-wise separable convolutions to reduce parameters and enhance computational efficiency; convolutional block attention modules to augment the model’s ability to focus on key features; multi-scale dilated convolution to expand the receptive field and capture multi-scale information; and bidirectional feature pyramid modules to achieve effective fusion and information flow of features at different levels. In tests on the Cornell dataset, our network achieved grasping pose prediction at a speed of 66.7 frames per second, with accuracy rates of 98.6% and 96.9% for image-wise and object-wise splits, respectively. The experimental results show that our method achieves high-speed processing while maintaining high accuracy. In real-world robotic grasping experiments, our method also proved to be effective, achieving an average grasping success rate of 95.6% on a robot equipped with parallel grippers.

## 1. Introduction

With the rapid advancement of science and technology, robotic technology has made significant strides across various fields. Among them, robotic arm grasping, as a crucial means of interaction between robots and their environment, has been widely applied in scenarios such as industrial production, warehousing logistics, and household services. However, numerous challenges persist in the practical implementation of robotic arm grasping. Firstly, the irregularity of objects to be grasped poses difficulties in detection, as objects of different shapes, sizes, and materials might require different grasping strategies. Secondly, the complexity of the grasping environment, such as changes in lighting and cluttered backgrounds, further complicates detection. Additionally, errors in grasp prediction are often significant, directly impacting the accuracy of subsequent grasping actions. These issues result in detection accuracy falling short of expectations, thereby affecting the success rate of the entire grasping task.

Traditional methods for robotic arm grasping are primarily based on the geometric features, physical models, and mechanical analysis of objects, achieving grasping operations through the establishment of grasping detection models [1,2]. However, these methods often struggle to meet the demands when confronted with complex, unstructured environments. In recent years, with the rapid rise of deep learning and computer vision technologies, researchers have begun to introduce these advanced techniques into the field of robotic arm grasping. Deep learning-based grasping methods [3,4,5,6,7,8,9,10,11,12,13,14,15,16,17] have quickly become mainstream in robotic grasping due to their advantages, such as no need for manual feature matching, strong feature extraction capabilities, and good robustness (see Section 2 for details). Nevertheless, existing grasp detection algorithms [3,4,5,6,7,8,9,10,11,12,13,14,16] struggle to achieve a perfect balance between accuracy and speed. Some algorithms excel in detection accuracy but are slow in execution, failing to meet the real-time requirements of robotic grasping. Others, while fast, lack the accuracy necessary to ensure precise grasping of target objects.

To address these issues, this paper proposes a new network for grasp pose prediction based on RGB-D data, termed the Cascaded Feature Fusion Grasping Network (CFFGN). This study aims to significantly enhance inference speed and computational efficiency while maintaining high accuracy through an innovative network architecture design. The main contributions of this paper include the following: proposing a novel multi-enhanced Cascaded Feature Fusion Grasping Network that effectively integrates RGB and depth information for efficient and accurate grasp pose estimation; introducing a Depth-wise Separable Convolution Module, significantly reducing the number of parameters and improving computational efficiency; employing a hybrid attention mechanism and dilated convolution module to enhance the model’s ability to perceive key features and expand its receptive field; and utilizing a bidirectional feature pyramid module to achieve effective fusion and information flow across different hierarchical features. Furthermore, we conducted extensive experiments on public datasets and real robotic platforms to validate the effectiveness and superiority of the proposed method.

The structure of this paper is as follows: first, we review related research work; then, we detail the proposed CFFGN network architecture and its key components; next, we present experimental results and performance evaluation; and finally, we summarize the findings of this research and discuss future research directions.

## 2. Related Work

The development of robotic grasp detection technology has undergone a transition from traditional methods to deep learning approaches. Geometric feature analysis identifies and matches the geometric shapes of objects to determine the optimal grasping posture, which is suitable for scenarios where object shapes are regular and predictable [1]. Physical models and mechanical analysis establish physical models between objects and the robotic arm, calculating grasping forces and points to ensure the stability and safety of the grasp. This is particularly crucial for controlling grasping force and torque [2].

In recent years, the advent of deep learning and computer vision technologies has prompted researchers to explore the integration of these advanced techniques into the domain of robotic grasping. The application of deep learning in the field of robotic grasping has rapidly become a mainstream approach due to the advantages it offers, including the capacity to extract features without the need for manual feature matching and its resilience. Lenz et al. [18] were the first to apply deep learning to the field of robotic grasp detection. A two-stage system was proposed, comprising a deep network for the initial identification of potential grasp points and a subsequent ranking of these points to facilitate the selection of the optimal grasp. Building on this, Redmon and Angelova [3] proposed a single-stage approach to real-time grasp detection utilizing convolutional neural networks (CNNs). The method they propose markedly enhances the velocity and precision of grasp detection, rendering it more compatible with practical robotic applications. Kumra and Kanan [4] further advanced the field by proposing a multimodal grasp detector that utilizes both RGB and depth information. The method achieved state-of-the-art performance on the Cornell grasp dataset through the utilization of transfer learning, whereby a ResNet network that had been pre-trained on ImageNet was fine-tuned. However, Levine et al. [5] employed a self-supervised learning approach, enabling the robot to learn from thousands of grasping attempts. This work demonstrates the potential of integrating deep learning with extensive real-world interaction data. Xu et al. [6] employed a single convolutional neural network to identify feasible robot grasps as oriented diameter circles in RGB images. The grasp representation is simplified by detecting the robot grasp as a directional-diameter circle. Morrison et al. [7] put forth a generative grasp convolutional neural network (GGCNN) for closed-loop real-time grasping, which is employed to forecast the grasp quality and pose of each pixel. Chu et al. [8] put forth a methodology wherein the grasp direction regression problem is transformed into a classification task, which is then combined with a grasp proposal network. This approach has the advantage of achieving efficient multi-object, multi-grasp detection. Ref. [19] put forth a novel transformer-based robot grasp detection method, designated TF-Grasp. An innovative application of the transformer was made to the robot grasp detection task. In comparison to a convolutional neural network (CNN), a transformer performs more effectively in establishing long-distance dependencies and learning powerful feature representations, which makes it a more suitable choice for grasp detection tasks in complex scenarios.

Recent developments in 2024 have further advanced the field through innovative architectural designs and feature enhancement mechanisms. Li et al. [12] introduced a lightweight model integrating large-kernel convolution with residual connections, achieving a 93.7% success rate in real-world testing while significantly reducing computational requirements. The challenge of cluttered environments has been addressed by Zhong et al. [13] through a novel two-cascade approach combining the Swin Transformer structure with Squeeze-and-Excitation attention mechanisms, achieving a 91.7% success rate in multi-target workspaces. Fang et al. [15] proposed LGAR-Net2. Further architectural innovations have emerged with the development of FAGD-Net by Zhong et al. [16] through its feature augmentation approach combining Residual Efficient Multi-Scale Attention with a Feature Fusion Pyramidal Module. Additionally, Kuang and Tao [17] introduced ODGNet, featuring omni-dimensional dynamic convolution. These recent advances demonstrate a clear trend toward lightweight architectures suitable for real-world deployment, robust performance in cluttered environments, and the successful integration of attention mechanisms for enhanced feature processing.

The aforementioned grasp detection algorithms each possess distinctive advantages and disadvantages, rendering it challenging to achieve an optimal equilibrium between accuracy and speed. Some algorithms demonstrate high accuracy in detection but are slow, which is not conducive to real-time grasping by robots. Other algorithms are rapid but lack accuracy, which presents a challenge in ensuring precise grasping of the target object by the robot. Consequently, there is a pressing need to develop new algorithms that can simultaneously address both detection accuracy and operating speed. Such algorithms will markedly enhance the performance of robots in practical applications, enabling them to complete grasping tasks with greater speed and precision.

## 3. Problem Statement

The issue of robotic two-fingered grasp detection can be defined as follows: Given an image of the object to be grasped in a given image *I*, the objective is to identify a successful grasp configuration *g*, whereby the grasp is performed vertically on a flat surface. The algorithm should be capable of mapping from the image *I* to the grasp configuration *g*.

(1) g=f(I)

**Figure 1:**
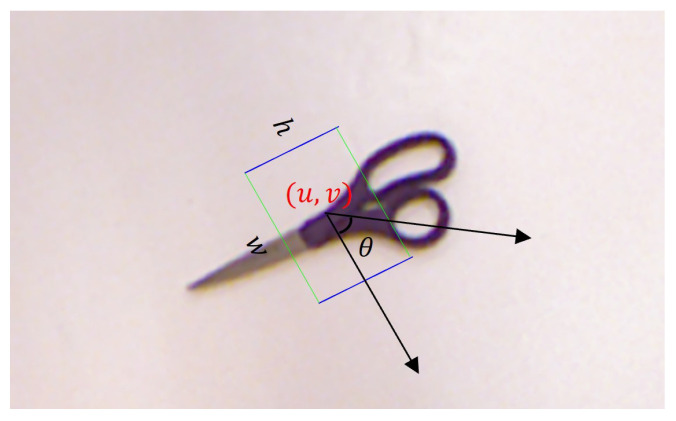
Grasping configuration representation.

In the spatial configuration of two-fingered grasping, the grasping configuration g can be represented by a five-dimensional parameter vector, as in Figure 1, which may be conceived of as a rotated rectangular box:

(2) g=(u,v,ω,h,θ)

where (u,v) represents the central coordinates of the rotating rectangular frame. A specific type of gripper is defined for grasping positions on the image plane. ω denotes the width of the rectangular frame. The opening width of the gripper determines the width of the graspable region. *h* represents the height of the rectangular frame, indicating the depth or thickness of the object in the grasping region, which corresponds to the gripper’s holding range. θ represents the rotation angle of the rectangular frame relative to the image coordinate system, which defines the gripper’s rotation direction and posture—specifically, the relative rotation angle of the gripper with respect to the object during grasping.

In the planar grasping process of robotic manipulation, the grasping pose is defined as follows:

(3) G=[p,Φ,w,Q]

where *G* represents the grasping pose matrix in the robot coordinate system, comprising the following parameters: *p* denotes the central coordinates of the gripper end-effector, Φ represents the rotation angle around the Z-axis of the gripper, *w* is the required grasping width, and *Q* represents the grasping quality score.

During the actual grasping process, the depth image $I\in R^{H\times W\times n}$ acquired by the robot’s sensor contains height *H* and width *W* information. For each pixel point *i* in the image space, its corresponding grasping pose is defined as follows:

(4) $$G_{i}=\left[\Phi_{i},w_{i},Q_{i}\right]$$

Specifically, the items denote the following:

- $\Phi_{i}$ represents the rotation angle for grasping.
- $w_{i}$ denotes the grasping width.
- $Q_{i}$ represents the grasping quality score for this position.

The grasping quality score is normalized within the range [0,1], where values approaching 1 indicate higher grasping quality, implying a higher probability of successful grasping. $\Phi_{i}$ describes the grasping angle executed at each point. Due to the gripper’s symmetry at $\pm \frac{\pi }{2}$, the angle range is defined as $\left[-\frac{\pi }{2},\frac{\pi }{2}\right]$. $w_{i}\in \left[0,W_{max}\right]$, where $W_{max}$ represents the maximum grasping width. The grasping set in the image space is defined as follows:

(5) $$G=\left[\Phi ,W,Q\right]\in R^{3\times H\times W}$$

where $\Phi ,W,Q\in R^{3\times H\times W}$, and each pixel point contains the following three values: $\Phi_{i},w_{i},Q_{i}$. This allows direct generation of grasping poses for each pixel in the image, from which the optimal grasping point $g^{*}$ can be found from the generated grasping set *G*:

(6) $$g^{*}=\max_{Q}G$$

**Figure 2:**
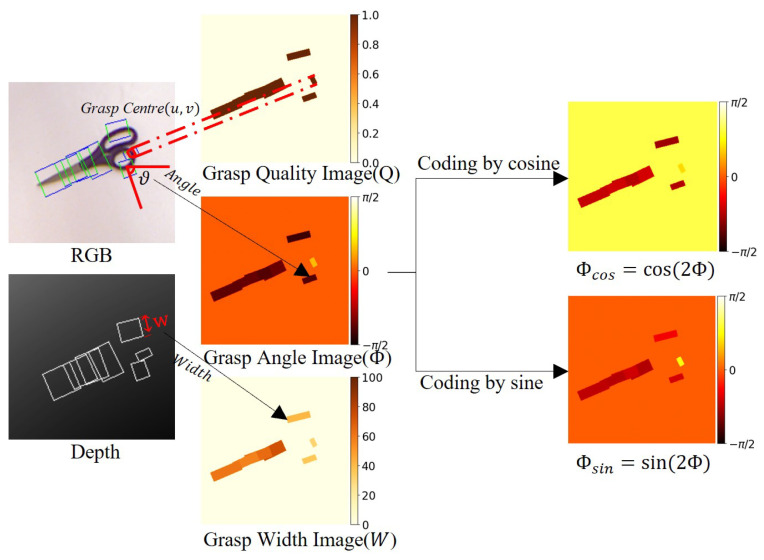
Illustration of the complete grasp representation and angle encoding pipeline. Left: input RGB-D images with grasp parameters annotated—grasp center (*u*, *v*), grasp angle (θ), and grasp width (*w*). Middle: three parameterized grasp maps derived from the input—the grasp quality map *Q* (values from 0 to 1.0, indicating grasp success probability), grasp angle map Φ (angle range $\left[-\frac{\pi }{2},\frac{\pi }{2}\right]$), and grasp width map W (in pixels). Right: angle encoding using trigonometric transformations—$\Phi_{cos}=\cos\left(2\Phi \right)$ and $\Phi_{sin}=\sin\left(2\Phi \right)$ to handle angle periodicity. The color scales indicate the range of values for each map—grasp quality (0–1.0), angles ($-\frac{\pi }{2}$ to $\frac{\pi }{2}$), and width (0–100 pixels).

As illustrated in Figure 2, our framework implements a comprehensive grasp representation pipeline. Starting from the left, the input consists of RGB and depth images from the Cornell dataset, where grasp parameters are explicitly annotated with grasp center (*u*, *v*), angle (θ), and width (*w*). These images undergo preprocessing operations including cropping and rotation. The framework then transforms these inputs into three essential parameterized maps (shown in the middle): (1) a grasp quality heat map *Q*, where values range from 0 to 1.0, indicating the probability of successful grasping at each pixel location; (2) a grasp angle heat map Φ, visualizing the optimal orientation for grasping within the range of $\left[-\frac{\pi }{2},\frac{\pi }{2}\right]$; and (3) a grasp width heat map *W*, representing the required gripper opening width in pixels for each potential grasp point. These maps together provide a complete pixel-wise representation of grasp configurations across the image space.

To address the periodic nature of grasp angles and ensure continuous representation, the angle map Φ is encoded using trigonometric functions. Specifically, we employ $\Phi_{cos}\;=\;\cos\left(2\Phi \right)$ and $\Phi_{sin}=\sin\left(2\Phi \right)$ transformations, which map the original angle range of $\left[-\frac{\pi }{2},\frac{\pi }{2}\right]$ to two complementary representations. This encoding scheme effectively handles angle periodicity and maintains the continuity of the angle representation.

For a robot to execute grasping actions in the image space, it is necessary to transform the image coordinates into the robot coordinate system. This transformation process can be realized through the following conversion matrix:

(7) $$G=T_{c}\left(T_{c}^{I}\left(G_{I}\right)\right)$$

where $T_{c}^{I}$ is the transformation matrix that converts the image coordinate system to the camera coordinate system and $T_{c}$ is the transformation matrix from the camera coordinate system to the robot coordinate system. During the actual grasping process, after obtaining the set of potential grasp poses *G* as defined in Equation (5), the optimal grasp pose g* can be determined through Equation (6), where g* represents the optimal grasp configuration selected from the grasp set *G* = [Φ, *W*, *Q* ] by finding the configuration with the highest quality score *Q*. This maintains consistency with our earlier definition, where *Q*$\in R^{3\times H\times W}$ represents the grasp quality scores for all pixel positions, and ensures we select the grasp pose that has the highest probability of successful execution by the robot.

## 4. Methodology

### 4.1. Overall Network Architecture

**Figure 3:**
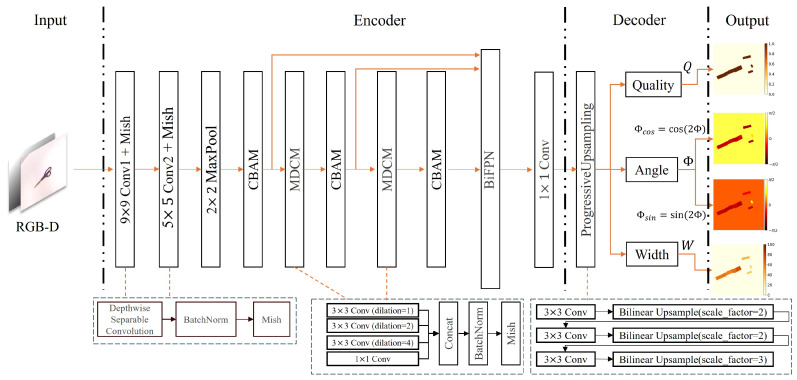
Network architecture of the Cascaded Feature Fusion Grasp Network (CFFGN).

This research proposes a Cascaded Feature Fusion Grasp Network (CFFGN) for grasp pose prediction based on RGB-D data. As shown in Figure 3, the network architecture accepts RGB images and depth maps as input. The feature extraction process employs a series of depth-wise separable convolutions, batch normalization, and Mish activation functions. The Channel and Block Attention Module (CBAM) is utilized to integrate channel attention with spatial attention, enabling the network to simultaneously consider channel and spatial dimensional feature redundancy.

Subsequently, the Multi-scale Convolution Dilated Module (MCDM) is employed to capture multi-scale information. The Bidirectional Feature Pyramid Network (BiFPN) is implemented for multi-scale feature fusion, incorporating progressive restoration of spatial feature resolution through gradient ascent. Finally, the network outputs information across four grasping channels.

### 4.2. Grasp Representation

Following Morrison et al. [7], we define the grasping collection in image space as $g=\left[\Phi ,W,Q\right]\in R^{3\times H\times W}$, where $\Phi ,W,Q\in R^{3\times H\times W}$, and each pixel point contains three values, $\phi_{i},w_{i},q_{i}$. These three values are described as follows:

*Q* represents the grasp quality at each pixel point in the image space. It indicates the success probability of grasping at that pixel point, and is a scalar value distributed in [0,1]. Values closer to 1 indicate higher grasping success rates. *W* represents the grasp width at each pixel point in the image space during grasping execution. To maintain constant width, the grasp width range is set to [0,100].

Φ represents the rotation angle at each pixel point during grasping execution. Since the dataset is annotated using image-level annotation, the predicted grasp pose is presented through a straight line. Therefore, the grasp angle range is $\left[-\frac{\pi }{2},\frac{\pi }{2}\right]$. We use cos(2×Φ) and sin(2×Φ) to predict the grasp angle. This method can solve the periodicity and discontinuity issues of angles, avoiding directional ambiguity while providing continuous and smooth representation. It maps angle information to the [−1,1] range, which is beneficial for neural network learning and optimization.

**Figure 4:**
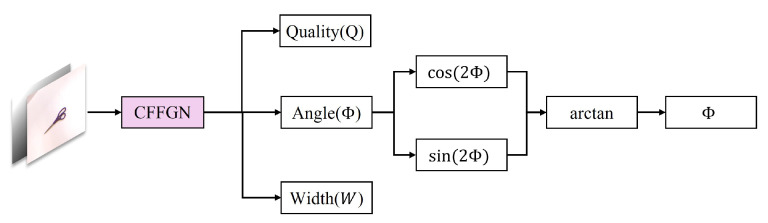
Grasp parameter calculation process. The network takes RGB-D data as input and outputs four values: *Q*, cos(2Φ), sin(2Φ), and *W*.

Meanwhile, this representation preserves complete directional information and considers the $180^{\circ }$ symmetry of objects in many grasping scenarios. As shown in Figure 4, the grasp parameter calculation process takes RGB-D data as input through network prediction, and outputs four values: *Q*, cos(2Φ), sin(2Φ), and *W*.

The rotation angle Φ is solved by

(8) $$\tilde{\Phi }=\frac{1}{2}\arctan\left(\frac{\sin(2\Phi )}{\cos(2\Phi )}\right)$$

### 4.3. Depth-Wise Separable Convolution Module

**Figure 5:**
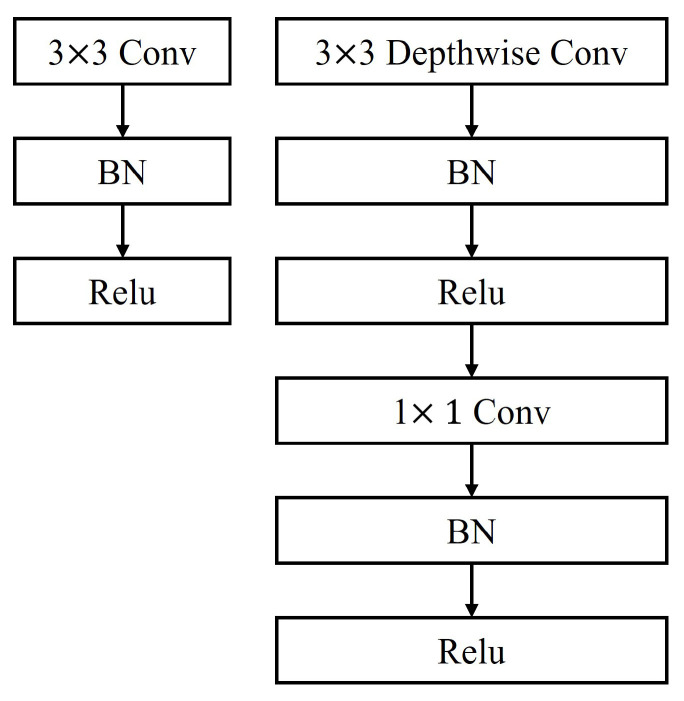
Left: standard convolution with BN and Relu layers.Right: depth-wise separable convolution structure.

To improve the computational efficiency while maintaining the representation capability of the CFFGN network, we introduced the Depth-wise Separable Convolution Module (DSCM), as shown in Figure 5. This module aims to reduce the network’s parameters and computational complexity while maintaining feature extraction capability. The core concept of the DSCM [20,21] is to decompose standard convolution operations into two independent steps: depth-wise convolution and point-wise convolution. This decomposition significantly reduces computational cost while preserving the model’s representational power.

The DSCM consists of two main steps: First is depth-wise convolution, which applies convolution operations independently to each input feature map channel, with each input channel having an independent convolution kernel. Assuming the convolution kernel size is k×k, the output *Z* of depth-wise convolution is calculated as follows:

(9) $$Z_{c,i,j}=\sum_{m=0}^{k-1}\sum_{n=0}^{k-1}X_{c,(i+m),(j+n)}\cdot W_{c,m,n}$$

where $Z_{c,i,j}$ is the value at channel *c* and position (i,j) in the output feature map. $X_{c,(i+m),(j+n)}$ is the value at channel *c* and position (i+m,j+n) in the input feature map *X*. $W_{c,m,n}$ is the weight at position (m,n) of the depth-wise convolution kernel *W* in channel *c*.

The second step is point-wise convolution, which uses a 1×1 convolution kernel to perform cross-channel information integration on the output of depth-wise convolution, enabling information flow and feature fusion between different channels. The output *Y* of point-wise convolution is calculated as follows:

(10) $$Y_{d,i,j}=\sum_{c=0}^{C-1}Z_{c,i,j}\cdot V_{d,c}$$

where $Y_{d,i,j}$ is the value at output channel *d* and position (i,j) in the final output feature map. $Z_{c,i,j}$ is the value at channel *c* and position (i,j) in the depth-wise convolution output feature map. $V_{d,c}$ is the weight between output channel *d* and input channel *c* in the point-wise convolution kernel.

Given input feature map $X\in R^{C\times H\times W}$, the operation of the DSCM can be expressed as follows:

(11) Y=PWConv(DWConv(X))

where DWConv(X) represents depth-wise convolution on feature map *X* and PWConv(Z) represents point-wise convolution on feature map *Z*, combining the output of depth-wise convolution across channels to obtain the final output feature map *Y*.

### 4.4. Multiple Enhanced Feature Extraction and Fusion

To enhance the network’s sensitivity to complex scene key features and achieve effective capture and fusion of multi-scale information, we introduced three key modules into the CFFGN network: the Convolutional Block Attention Module (CBAM) [22], Multi-scale Cross-dimensional Dilated Module (MCDM) [23], and Bidirectional Feature Pyramid Network (BiFPN) [24].

The CBAM enhances the network’s attention to important features by combining channel attention and spatial attention mechanisms. Specifically, the channel attention mechanism utilizes global average pooling and max pooling to generate channel descriptors, while spatial attention learns inter-channel relationships through multiple receptors to create a three-dimensional spatial attention map, highlighting areas rich in information. This dual attention mechanism enables the network to more accurately locate and identify potential grasp points.

Following CBAM, the MCDM employs convolutions with different dilation rates, effectively expanding the receptive field and enabling the network to capture multi-scale hierarchical information. By parallel processing using convolutions with different dilation rates, the MCDM can simultaneously handle objects of varying sizes and shapes, improving the network’s adaptability to complex scenes.

Finally, the BiFPN module serves as an efficient feature fusion network, enabling bidirectional information flow between different layer features. Compared to traditional feature pyramid networks, the BiFPN introduces additional cross-layer connections and adaptive feature fusion weights, allowing more efficient exchange and fusion between high-level semantic information and low-level detail features. This design not only improves feature utilization efficiency but also enhances the network’s ability to detect objects at different scales.

Through the collaborative effect of these three modules, the CFFGN network can extract richer and more distinctive features from the input RGB-D data, providing a solid foundation for subsequent grasp pose prediction. Experimental results demonstrate that this multi-dimensional enhancement of feature extraction and fusion strategy significantly improves network performance, enabling accurate and stable grasp prediction across various complex scenarios.

#### 4.4.1. Convolutional Block Attention Module (CBAM)

**Figure 6:**
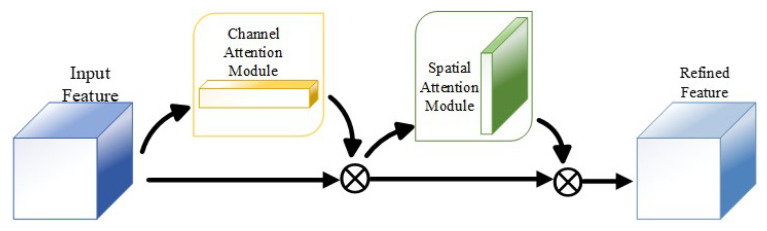
Schematic diagram of the CBAM module. This module comprises two components: the channel attention module and the spatial attention module. The input features undergo sequential processing.

The Convolutional Block Attention Module (CBAM) enhances the network’s feature representation capability through the integration of channel and spatial attention mechanisms [22]. This module effectively improves the network’s ability to capture important features, as illustrated in Figure 6.

The input features first pass through the channel attention module, which emphasizes significant channel information. Subsequently, the spatial attention module processes the output, highlighting key spatial locations. This sequential process results in refined feature maps.

**Figure 7:**
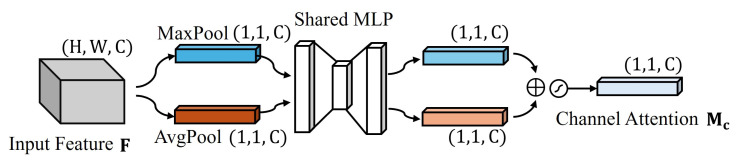
Channel attention module in the CBAM. The input feature F with dimensions H × W × C undergoes global max pooling (MaxPool) and average pooling (AvgPool) operations, resulting in two feature descriptors of size 1 × 1 × C. These descriptors are then processed by a shared multi-layer perceptron (Shared MLP). The outputs are combined to generate the final channel attention map $M_{c}$ with dimensions 1 × 1 × C.

In the channel attention mechanism, as shown in Figure 7, the input feature map initially undergoes global average pooling and global max pooling operations. The resulting outputs are defined as follows:

(12) $$F_{avg}^{c}=\frac{1}{H\times W}\sum_{i=1}^{H}\sum_{j=1}^{W}F^{c}\left(i,j\right)$$

(13) $$F_{max}^{c}=\max_{i,j}F^{c}\left(i,j\right)$$

where $F^{c}$ represents the *c*-th channel of the input feature and *H* and *W* denote the height and width of the feature map, respectively. These two feature descriptors are processed by shared multi-layer perceptrons to generate the channel attention map:

(14) $$M_{c}\left(F\right)=\sigma \left(W_{1}\left(W_{0}\left(F_{avg}^{c}\right)\right)+W_{1}\left(W_{0}\left(F_{max}^{c}\right)\right)\right)$$

where $W_{0}\in R^{C/r\times C}$, $W_{1}\in R^{C\times C/r}$, *C* is the number of channels in the input feature, *r* is the reduction ratio, and σ denotes the sigmoid activation function.

As illustrated in Figure 7, the channel attention module of the CBAM processes the input feature F (H × W × C) through global max pooling (MaxPool) and average pooling (AvgPool) operations. This results in two feature descriptors of size 1 × 1 × C. These descriptors are then processed by a shared multi-layer perceptron (Shared MLP). The outputs are combined to produce the final channel attention map $M_{c}$ with dimensions 1 × 1 × C.

**Figure 8:**
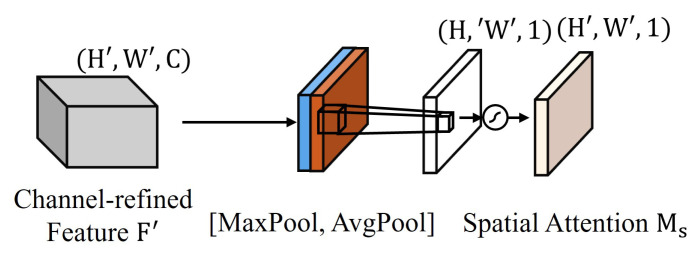
Spatial attention module in the CBAM. The channel-refined feature F′ with dimensions H′ × W′ × C undergoes max pooling and average pooling operations, resulting in features of size H′ × W′ × 1. These are then processed to generate the spatial attention map $M_{s}$ with dimensions H′ × W′ × 1, which captures important spatial information in the input feature map.

The spatial attention mechanism, depicted in Figure 8, operates as follows:

(15) $$F_{avg}^{s}=\frac{1}{C}\sum_{c=1}^{C}F^{c}$$

(16) $$F_{max}^{s}=\max_{c}F^{c}$$

After channel-wise pooling, a 7 × 7 convolution is applied to generate the spatial attention map:

(17) $$M_{s}\left(F^{\prime }\right)=\sigma \left(f^{7\times 7}\left(\left[F_{avg}^{s};F_{max}^{s}\right]\right)\right)$$

where $f^{7\times 7}$ denotes the 7 × 7 convolution operation and [;] represents channel concatenation. The final output of CBAM is given by

(18) $$F^{\prime \prime }=M_{s}\left(M_{c}\left(F\right)⊗F\right)⊗\left(M_{c}\left(F\right)⊗F\right)$$

where ⊗ represents element-wise multiplication.

#### 4.4.2. Multi-Scale Dilated Convolution Module (MCDM)

**Figure 9:**
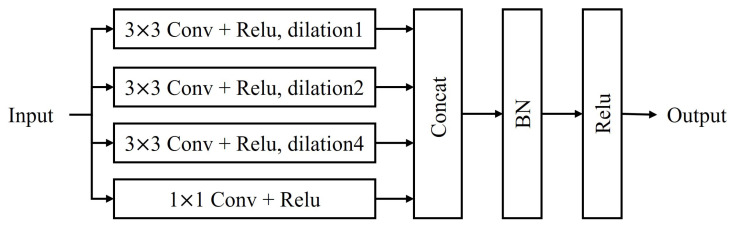
Structure of the Multi-scale Dilated Convolution Module (MCDM).

The Multi-scale Dilated Convolution Module (MCDM) utilizes convolutions with different dilation rates to capture multi-scale spatial information and expand the receptive field [23]. The mathematical expression for dilated convolution is given by

(19) $$y\left[i\right]=\sum_{k}x\left[i+r\cdot k\right]\cdot w\left[k\right]$$

where *x* is the input, *w* is the convolution kernel, *r* is the dilation rate, and *y* is the output. The MCDM comprises four parallel convolution branches: a 1 × 1 standard convolution and three 3 × 3 dilated convolutions (*r* = 1, 2, 4). These branches have effective receptive fields of 1 × 1, 3 × 3, 5 × 5, and 9 × 9, respectively, as illustrated in Figure 9. The outputs of these branches are combined as follows:

(20) $$Y=concat[Conv_{1}\left(X\right),Conv_{2}\left(X\right),Conv_{3}\left(X\right),Conv_{4}\left(X\right)]$$

where $Conv_{i}$ represents the *i*-th convolution branch, *X* is the input feature map, and concat denotes the concatenation operation along the channel dimension. This architecture allows the network to simultaneously capture features at different scales.

As shown in Figure 9, the MCDM consists of the following:

- Three 3 × 3 convolutions with ReLU activation and different dilation rates (1, 2, and 4).
- One 1 × 1 convolution with ReLU activation.
- A concatenation layer to combine the outputs of all branches.
- A batch normalization (BN) layer.
- A final ReLU activation.

This module effectively integrates multi-scale features, enhancing the network’s ability to capture spatial information at various scales.

#### 4.4.3. Bidirectional Feature Pyramid Network (BiFPN)

**Figure 10:**
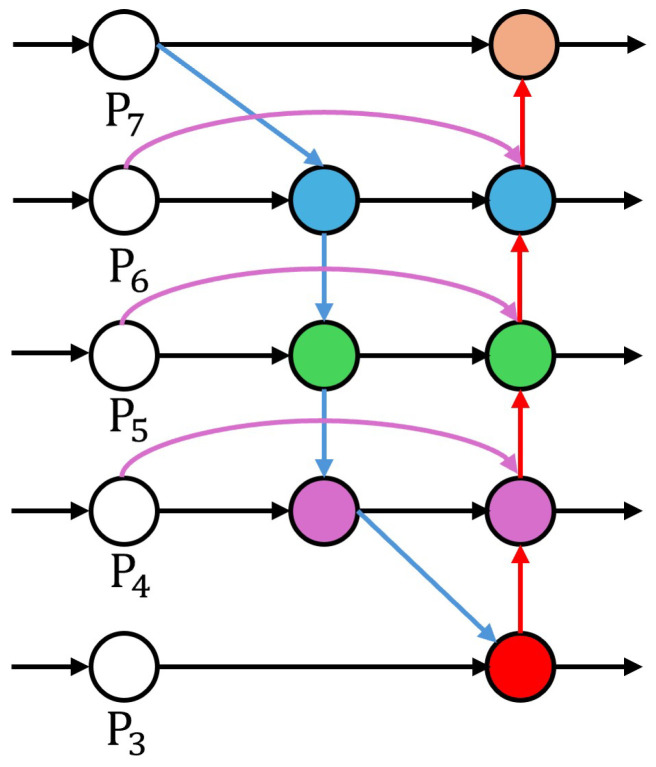
BiFPN structure diagram. P3–P7: represent feature maps of different scales, from the shallow layer (P3) to the deep layer (P7). Red arrows: top-down path, fusing high-level semantic information to low-level features. Blue arrows: bottom-up path, propagating fine-grained information from low-level to high-level features. Purple arrows: same-level connections, integrating features from the same scale. Black arrows: flow paths of the initial features. The colored circles in the diagram represent feature maps at different scales. From P3 to P7, they indicate feature maps progressing from the shallow layer (P3) to the deep layer (P7).

The Bidirectional Feature Pyramid Network (BiFPN) integrates multi-scale feature information through bidirectional (top-down and bottom-up) information flow and fast feature fusion [24]. As shown in Figure 10, it includes seven feature applications, with a structure comprising both top-down and bottom-up paths. The mathematical representations are as follows:

(21) $$P_{i}^{td}=Conv_{3\times 3}\left(w_{1}^{i}P_{i}^{in}+w_{2}^{i}Resize\left(P_{i-1}^{td}\right)\right)$$

(22) $$P_{i}^{out}=Conv_{3\times 3}\left(w_{1}^{i}P_{i}^{in}+w_{2}^{i}P_{i}^{td}+w_{3}^{i}Resize\left(P_{i+1}^{out}\right)\right)$$

where $P_{i}^{in}$, $P_{i}^{td}$, and $P_{i}^{out}$ represent the input feature, top-down intermediate feature, and final output feature at level *i*, respectively. $w_{i}^{j}$ are learnable weights, the Resize() operation ensures consistent feature map dimensions, and $Conv_{3\times 3}$ denotes a 3 × 3 convolution operation.

The BiFPN incorporates a fast normalization technique to calculate weights, which is defined as follows:

(23) $$w_{i}=\frac{a_{i}}{\epsilon +\sum_{j}a_{j}}$$

where $a_{i}$ are learnable parameters and ϵ=0.0001 is used for numerical stability. Assuming there are *L* levels of features, each feature map has dimensions $W_{i}\times H_{i}\times C_{i}$.

Figure 10 illustrates the BiFPN structure. The red arrows indicate the top-down path, which fuses high-level semantic information into low-level features. The blue arrows represent the bottom-up path, propagating fine-grained information from low-level to high-level features. Purple arrows denote same-level connections, integrating features from the same scale. Black arrows represent the initial features of flow paths.

#### 4.4.4. Integration of Multiple Modules in CFFGN

The Composite Feature Fusion and Guidance Network (CFFGN) leverages the synergistic effects of three key modules to extract richer and more discriminative features from the input RGB-D data. The integration of these modules enhances the network’s overall performance and adaptability across various scenarios:

- CBAM (Convolutional Block Attention Module): This module strengthens the network’s focus on the most relevant features. By implementing both channel and spatial attention mechanisms, the CBAM enhances the network’s ability to capture crucial information within the input data.
- MCDM (Multi-scale Dilated Convolution Module): By employing convolutions with various dilation rates, the MCDM enables the network to capture multi-scale information effectively. This approach allows for a comprehensive understanding of features at different spatial scales, contributing to improved feature representation.
- BiFPN (Bidirectional Feature Pyramid Network): This module facilitates the efficient fusion of features across different scales. Through its bidirectional information flow, the BiFPN enables the network to integrate both high-level semantic information and low-level fine-grained details, resulting in a more robust feature representation.

The combined application of these modules in CFFGN results in a feature extraction and fusion process that is both comprehensive and refined. This integrated approach forms the foundation for accurate depth estimation, significantly enhancing the network’s capability to perform effectively across diverse scenarios and environmental conditions.

By leveraging the strengths of each module, CFFGN achieves a synergistic effect that surpasses the capabilities of individual components. This holistic approach to feature processing and fusion contributes to the network’s improved performance in depth estimation tasks, demonstrating its adaptability and robustness in various real-world applications.

### 4.5. Experiment Conditions

The CFFGN model was trained and tested on the Ubuntu 22.04 operating system. The hardware configuration consisted of an Intel^®^ Xeon(R) Gold 5118 CPU @ 2.30 GHz × 48 and an NVIDIA TITAN Xp GPU. The system was equipped with 128 GB of RAM. The software environment utilized the PyTorch 2.0 deep learning framework with Python 3.9.

#### 4.5.1. Training Strategy and Parameter Selection

The training parameters were carefully selected based on extensive preliminary experiments and ablation studies:

- Number of epochs: 200 (determined by monitoring validation loss convergence);
- Batch size: 32 (optimized for GPU memory utilization and training stability);
- Optimizer: Adam (chosen for its adaptive learning rate capability and robust performance);
- Initial learning rate: 0.001 (with cosine annealing scheduler);
- Weight decay: 1 × 10^−4^ (to prevent overfitting);
- Early stopping patience: 20 epochs.

To ensure stable training and prevent gradient explosion, we employed gradient clipping with a maximum norm of 1.0. The learning rate was managed using a cosine annealing scheduler, which gradually reduces the learning rate following a cosine curve pattern. This approach helps the model converge to better local optima while maintaining training stability.

#### 4.5.2. Model Generalization Strategies

Several techniques were implemented to enhance the model’s generalization ability:

- Dropout layers: incorporated with a rate of 0.3 in the fully connected layers to prevent overfitting;
- Batch normalization: applied after convolutional layers to stabilize training and improve generalization;
- Data augmentation: implemented various transformation techniques including random rotation (±30°), random cropping (maintaining 85% of original image), and horizontal flipping (50% probability) to increase data diversity and simulate real-world variations.

### 4.6. Dataset and Preprocessing

We employed two widely used benchmark datasets to train and evaluate the CFFGN model: the Cornell dataset and the Jacquard dataset. The Cornell dataset, developed by Saxena et al. from Cornell University, contains 885 RGB-D images of 240 distinct household objects captured in various poses and configurations. The dataset was systematically constructed using a Kinect-style sensor mounted at a fixed height above a uniform background table surface. Objects were placed in different orientations to capture multiple views, encompassing a diverse range of household items such as kitchen utensils, tools, and office supplies, representing both regular (boxes and cylinders) and irregular shapes.

Each RGB-D image pair in the Cornell dataset consists of one 640 × 480 resolution RGB image in PNG format and one corresponding depth image in PCD format. The dataset includes 5–10 manually labeled grasp rectangles per image, totaling 8019 labeled grasps across the dataset. These grasp annotations were carefully created by human experts and verified through physical robot experiments. Each grasp is defined by five parameters (x,y,θ,h,w), where (x,y) represents the grasp center position, θ indicates the grasp angle, and (h,w) specify the height and width of the grasp rectangle.

The Jacquard dataset, a more recent and comprehensive collection, contains over 54,000 RGB-D images of more than 11,000 unique objects, representing a significant expansion in scale compared to the Cornell dataset. Each image in the Jacquard dataset is accompanied by both successful and unsuccessful grasp annotations, validated through extensive simulation using the Bullet Physics engine. The dataset features objects with varying geometries, textures, and material properties, captured under different lighting conditions and viewpoints. The images are recorded using a combination of structured light and stereo vision systems, providing high-quality depth information with a resolution of 1024 × 1024 pixels.

While both datasets have proven invaluable for robotic grasping research, they carry certain limitations. The Cornell dataset is limited to single-object scenarios, uniform background and lighting conditions, and lacks highly reflective or transparent objects, focusing primarily on parallel-jaw gripper configurations. The Jacquard dataset, while more diverse, may still exhibit simulation-to-reality gaps in grasp success validation, and its synthetic nature can sometimes lead to less realistic object interactions compared to real-world scenarios. Despite these constraints, the complementary nature of these datasets provides a robust foundation for training and evaluating grasping algorithms.

### 4.7. Assessment Indicators

To evaluate the detection performance of the CFFGN model, we employed the rectangle-based metric IoU (Intersection over Union) [9]. IoU is a crucial evaluation metric for object detection tasks, measuring the degree of overlap between the predicted bounding box and the ground truth bounding box. The mathematical formula for IoU is as follows:

(24) $$IoU=\frac{\left|A\cap B\right|}{\left|A\cup B\right|}=\frac{\left|A\cap B\right|}{\left|A|+|B|-|A\cap B\right|}$$

where

- *A* represents the area of the predicted bounding box;
- *B* represents the area of the ground truth bounding box;
- |A∩B| denotes the intersection area of *A* and *B*;
- |A∪B| denotes the union area of *A* and *B*;
- |A| represents the area of *A*;
- |B| represents the area of *B*.

For the Cornell dataset, we adopted the Jaccard index (IoU) to determine whether the predicted grasp is correct. Specifically, a prediction is considered correct if it satisfies two conditions:

1. The IoU between the predicted bounding box and the ground truth bounding box is greater than 25%.
2. The angle error between the predicted grasp and the ground truth grasp is less than 30°.

These criteria ensure that the model’s predictions are both spatially accurate and correctly oriented, which is crucial for successful robotic grasping tasks. By using these stringent evaluation metrics, we can comprehensively assess the performance of our CFFGN model in terms of both localization accuracy and orientation precision.

### 4.8. Experiments

#### 4.8.1. Performance on Cornell Dataset

To validate the performance of CFFGN, we conducted extensive experiments on the Cornell dataset. We employed a cross-validation approach to evaluate CFFGN, with the final results reported as the average of five independent trials. Furthermore, we utilized two distinct data splitting methods:

- Image-wise split: randomly selecting 80% of the images for training and the remaining 20% for testing.
- Object-wise split: ensuring that objects in the training set do not appear in the test set.

These splitting strategies allow for a comprehensive assessment of the model’s ability to generalize to both unseen images and novel objects, evaluating its robustness in handling various object positions and pose variations.

As shown in Table 1, CFFGN achieved impressive performance.

The model demonstrated high accuracy in both splitting scenarios, with 98.6% for image-wise and 96.9% for object-wise splits. Moreover, CFFGN achieved a detection speed of 15 ms, surpassing other algorithms. These results underscore CFFGN’s ability to maintain high detection accuracy while ensuring real-time performance.

The experimental outcomes across different splitting methods indicate that CFFGN exhibits robust generalization ability in handling various object positions and pose variations. Additionally, the model demonstrates strong transfer learning capabilities when dealing with previously unseen objects.

In comparison with existing methods, Morrison et al.’s approach [7] achieved the lowest parameter count but suffered from lower detection accuracy. Conversely, other methods attained higher accuracy but at the cost of increased parameter counts. Our proposed CFFGN strikes a balance with a parameter count of 0.104 M, exemplifying the model’s efficiency. This lightweight design enables CFFGN to maintain both high detection accuracy and real-time processing speed, showcasing its comprehensive performance in both aspects.

#### 4.8.2. Performance on Jacquard Dataset

To further validate CFFGN’s effectiveness, we conducted extensive experiments on the larger-scale Jacquard dataset. Following standard protocols, we utilized an 80:20 train/test split ratio for evaluation. Table 2 presents the comparative results.

CFFGN achieved state-of-the-art performance with 96.5% accuracy while maintaining its efficient parameter count of 0.104 M and real-time detection speed of 14 ms. The model’s superior performance on this larger, more diverse dataset demonstrates its robust generalization capabilities and effectiveness in handling various object geometries and grasping scenarios. Notably, CFFGN outperformed more complex architectures like [26,27], validating our lightweight design approach for efficient robotic grasping.

The consistent performance across both Cornell and Jacquard datasets underscores CFFGN’s versatility and reliability in diverse grasping scenarios, making it particularly suitable for real-world robotic applications where both accuracy and computational efficiency are crucial.

#### 4.8.3. Ablation Experiments

To validate the effectiveness of each key component in the CFFGN model, we conducted a series of ablation studies. These experiments aimed to analyze the contribution of each module to the overall performance of the model, including the Depth-Separable Convolution Module (DSCM), Convolutional Block Attention Module (CBAM), Multi-scale Dilated Convolution Module (MCDM), and Bidirectional Feature Pyramid Network (BiFPN).

**Figure 11:**
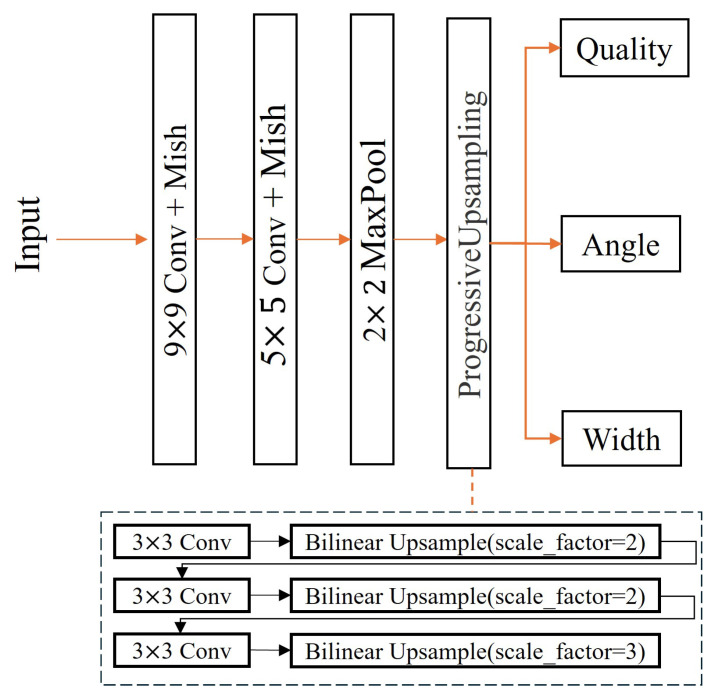
Architecture of the Baseline network. It consists of a 9 × 9 convolutional layer, followed by 5 × 5 and 2 × 2 max pooling layers, and then progressive upsampling layers.

In our ablation experiments, we used a Baseline network as the foundation of CFFGN, which does not include any of our proposed high-level modules. The Baseline network is a relatively simple structure, primarily based on the standard convolutional neural network architecture, as shown in Figure 11. It employs basic convolution, pooling, and upsampling operations but does not incorporate our proposed advanced modules such as the DSCM, CBAM, MCDM, and BiFPN.

The Baseline network is designed to provide a performance benchmark, allowing us to quantify the improvements brought by each new module. By comparing with this Baseline, we can clearly evaluate the contribution of each component to the network’s overall performance.

Figure 11 illustrates the structure of the Baseline network. The input passes through a 9 × 9 convolutional layer, followed by a 5 × 5 convolutional layer and a 2 × 2 max pooling layer. The network then employs a progressive upsampling module to produce the final output.

The progressive upsampling module, shown in the lower part of the figure, consists of three parallel paths:

- The first two paths each contain a 3 × 3 convolutional layer followed by a bilinear upsampling layer with a scale factor of 2.
- The third path includes a 3 × 3 convolutional layer followed by a bilinear upsampling layer with a scale factor of 3.

This progressive upsampling approach allows the network to gradually increase the spatial resolution of the feature maps while incorporating information from different scales. The outputs of these upsampling paths are then combined to produce the final feature map.

The network’s output includes three branches for predicting the quality, angle, and width of the grasp. This Baseline architecture, with its progressive upsampling module, serves as the foundation for our ablation studies, allowing us to evaluate the impact of additional advanced modules on the network’s performance.

We conducted ablation experiments on the Cornell dataset, using both image-wise split and object-wise split methods. We recorded the detection accuracy and speed for each configuration. Table 3 and Table 4 present the results of these ablation studies.

Through analysis of the experimental results in Table 3 and Table 4, we can better understand the impact of each module on the performance of the CFFGN model and its underlying mechanisms. First, from the single-module ablation experiment results, we can see that the introduction of the Depth-Separable Convolution Module (DSCM), while providing a relatively limited improvement in accuracy (0.36% increase for image-wise and 1.12% for object-wise), significantly reduced the model’s computational complexity, improving detection speed from 30 ms to 11 ms. This is because the DSCM decomposes standard convolution into depth-wise and point-wise convolutions, greatly reducing the number of parameters and computations while maintaining good feature extraction capabilities.

The introduction of the Convolutional Block Attention Module (CBAM) brought considerable performance improvements, with image-wise and object-wise accuracies increasing by 2.78% and 5.54%, respectively. This improvement stems from the CBAM’s ability to adaptively adjust feature map weights, both highlighting important feature maps in the channel dimension and focusing on key areas in the spatial dimension, thus enhancing the model’s ability to perceive grasp point features. The significant improvement in object-wise split indicates that the CBAM enhanced the model’s generalization ability for different objects.

The Multi-scale Dilated Convolution Module (MCDM), by expanding the receptive field without increasing the number of parameters, enables the model to capture multi-scale contextual information, which is particularly effective for detecting grasp positions of different sizes. Experimental results show that the MCDM brought accuracy improvements of 3.72% and 6.43%, while only slightly increasing computation time (from 30 ms to 33 ms). This good balance between performance and efficiency confirms the superiority of the MCDM in feature extraction.

The Bidirectional Feature Pyramid Network (BiFPN), when used as an individual module, brought the most significant accuracy improvements (4.59% increase for image-wise and 7.25% for object-wise). The BiFPN achieves efficient fusion of multi-scale features through top-down and bottom-up bidirectional connections, enhancing the model’s ability to detect grasp targets at different scales. This feature fusion mechanism is particularly helpful in improving the model’s robustness in complex scenarios.

In the multi-module combination experiments, significant synergistic effects were demonstrated between the modules. The combination of the CBAM and MCDM (accuracy improvements of 6.18% and 8.48%) shows that the integration of attention mechanisms and multi-scale feature extraction can mutually promote each other, both highlighting important features and ensuring feature diversity. The combination of the CBAM and BiFPN produced the best effect (accuracy improvements of 6.70% and 9.04%), indicating that attention-assisted feature selection can further enhance the fusion effect of the feature pyramid. The combination of three modules (CBAM + MCDM + BiFPN) achieved the most significant performance improvement (accuracy increases of 8.60% and 11.18%), although detection time increased to 44ms, balancing precision and efficiency.

The final complete CFFGN model not only achieved the highest accuracy (98.62% and 96.91%) but also realized the fastest detection speed (15 ms). This qualitative leap in performance is due to the complementary and synergistic effects of each module at different levels: the DSCM provides efficient basic feature extraction, the CBAM realizes the highlighting of key features, and the MCDM ensures the acquisition of multi-scale information, while BiFPN optimizes hierarchical feature fusion. This multi-level, multi-faceted feature optimization strategy ultimately enables CFFGN to achieve significant improvements in both accuracy and efficiency.

### 4.9. Actual Robot Grasping Experiments

To validate the performance of the proposed algorithm in real-world settings, we constructed a robotic grasping experimental platform. This platform primarily consists of three components: the robot body, a vision system, and an end-effector. The robot utilizes an EPSON C4-A901S six-axis robotic arm, which offers high positioning accuracy and flexibility. The vision system employs an Intel RealSense D415 depth camera, mounted above the workspace in an “eye-to-hand” configuration to provide a broader field of view. The end-effector is an electric parallel two-finger gripper, capable of handling objects of various shapes and sizes.

**Figure 12:**
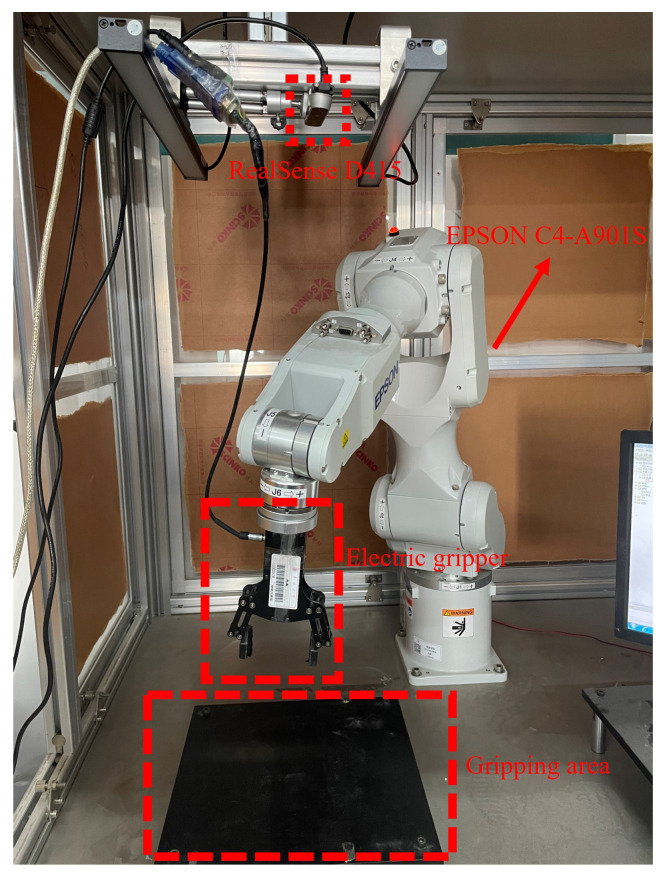
Experimental platform setup for robotic grasping. The platform integrates an EPSON C4-A901S six-axis robot arm equipped with an electric parallel gripper as the end-effector. A RealSense D415 depth camera is mounted overhead in an eye-to-hand configuration. The gripping area (marked with red dashed box) represents the workspace where objects are placed for grasping experiments. All key components are labeled for clarity.

Figure 12 illustrates our experimental platform setup in detail. The system is constructed within an aluminum profile frame structure that provides stability and flexibility for component mounting. The RealSense D415 depth camera is fixed at the top of the frame, approximately 1 m above the workspace, ensuring a comprehensive view of the gripping area. This eye-to-hand configuration allows the camera to capture both the RGB and depth information of the entire workspace without interference from the robot’s movements.The EPSON C4-A901S six-axis robot arm is securely mounted on one side of the platform, with its workspace centered on the gripping area (highlighted by the red dashed box). The electric parallel gripper attached to the robot’s end-effector is specifically designed for precise grasping tasks, featuring an adjustable gripping force and width to accommodate various object sizes. The entire setup is optimized to ensure good visibility for the vision system while maintaining maximum reachability for the robot arm within the designated gripping area.

The experimental procedure mainly includes the following steps: First, the depth camera captures RGB-D images of the workspace. Then, the CFFGN algorithm proposed in this paper is executed to perform grasp detection, obtaining the optimal grasp position. Next, the grasp position is transformed to the robot’s base coordinate system. Finally, the robot controller executes the grasping motion.

To comprehensively evaluate the algorithm’s performance, we randomly selected 10 common household items as grasping targets. These items include fruits, stationery, and other objects with varying shapes, sizes, and materials. It is worth noting that all experimental objects were previously unseen during training, which helps to test the algorithm’s generalization ability.

**Figure 13:**
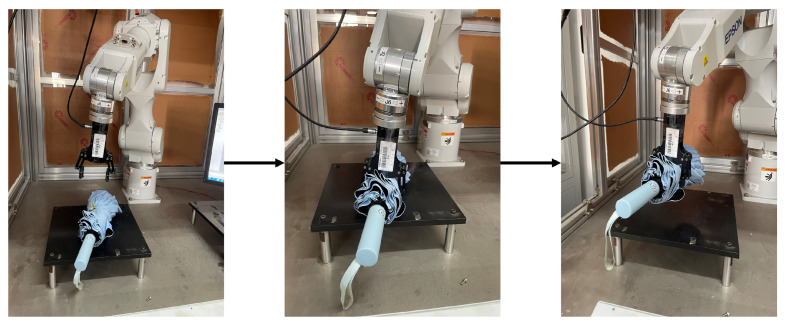
Sequential demonstration of a successful umbrella grasping experiment. Left: the robotic arm approaches the target umbrella based on the predicted optimal grasping pose. Center: the gripper aligns with the detected grasping point on the umbrella body and adjusts to the appropriate width. Right: the gripper successfully executes the grasp and lifts the umbrella, demonstrating the algorithm’s capability to identify and execute grasps on the main body structure rather than conventional grasping points like handles.

Figure 13 demonstrates a complete grasping sequence using an umbrella as an example. The process consists of three key stages: approach, alignment, and execution. As shown in the sequence, our algorithm successfully identifies a suitable grasping point on the umbrella’s folded fabric body rather than the conventional handle location. This demonstrates the algorithm’s ability to analyze object geometry and find stable grasping points even when they are not at obvious or traditional grasping locations. The successful grasp and lift of the umbrella from its main body validates both the effectiveness of our grasping pose prediction and the algorithm’s capability to identify novel grasping solutions for everyday objects.

In the experiment, we attempted 25 grasps for each object, with the object’s position and pose randomly changed for each attempt. We recorded the success and failure of each grasp and calculated the overall success rate. The experimental results are shown in Table 5 below.

Analysis of the experimental data reveals that the CFFGN algorithm demonstrates high stability and effectiveness across various object grasping tasks. The average success rate of 95.6% underscores the algorithm’s potential in practical application scenarios. Notably, for objects with regular geometric shapes, such as milk cartons, pens, small parcel boxes, and batteries, the algorithm achieved a 100% grasping success rate. This result can be attributed to the relatively simple shape features of these objects, which facilitate accurate grasp point localization and pose prediction.

In contrast, the grasping success rate for watches was 88%, slightly lower than for other objects, but still maintaining a high level of performance. This discrepancy may stem from the complex geometric structure and potentially reflective surfaces of watches, presenting additional challenges for visual perception and grasp planning. Nevertheless, even when faced with such intricate objects, the algorithm exhibited satisfactory performance, further confirming its robustness.

For items that may exhibit dynamic instability, such as apples, the algorithm also performed exceptionally well, achieving a 96% success rate. This result highlights the CFFGN algorithm’s adaptability in handling potentially unstable objects, which is of significant importance for grasping tasks in real-world application environments.

## 5. Conclusions

This paper addresses the efficiency and accuracy challenges in robotic grasping of complex shapes and diverse objects by proposing a novel Cascaded Feature Fusion Grasping Network (CFFGN) based on RGB-D data. By incorporating depth-wise separable convolutions, hybrid attention mechanisms, multi-scale dilated convolutions, and bidirectional feature pyramid modules, the model significantly enhances computational efficiency, feature perception capabilities, and multi-scale information capture. In both the Cornell dataset and real-world robotic experiments, CFFGN demonstrated superior performance, achieving rapid grasp pose prediction while maintaining high accuracy, thus meeting the requirements for real-time robotic grasping.

Experimental results indicate that CFFGN possesses strong generalization capabilities and robustness in complex, dynamic grasping environments, maintaining high success rates even when confronted with previously unseen objects. However, this study has some limitations, such as suboptimal performance in handling transparent or reflective objects, which may be related to the precision of depth information acquisition.

Future research directions will focus on the following aspects:

1. Further optimization of the network structure to enhance model robustness in processing complex object shapes and materials;
2. Exploration of multi-sensor fusion strategies, incorporating perceptual information beyond RGB-D to address challenges posed by transparent or reflective objects;
3. Consideration of applying this network to larger-scale multi-object grasping scenarios and improving adaptability to complex tasks.

## Figures and Tables

**Table 1:** Comparative results on Cornell dataset using different splitting methods.

Network	Accuracy (%)		Parameter ^1^	Detection ^2^
Model	Image-Wise	Object-Wise	Count (M)	Speed (ms)
[9]	60.5	58.3	-	5000
[3]	80.1	87.1	-	76
[4]	89.2	88.9	⩾32	102.9
[7]	73.0	69.0	0.062	19
[11]	93	-	18	800
[5]	80	-	1	200
[8]	96.0	96.1	28.18	120
[10]	92.4	90.7	3.71	24
[12]	97.0	96.5	0.6	15
[13]	97.8	-	-	40
[14]	98.2	97.1	17.23	25
[16]	96.5	-	-	23
CFFGN (Ours)	98.6	96.9	0.104	15

^1^ Parameter count is in millions (M). ^2^ Detection speed is in milliseconds (ms).

**Table 2:** Comparative results on Jacquard dataset.

Network	Accuracy (%)	Parameter Count (M)	Detection Speed (ms)
[15]	94.4	-	17
[7]	84	0.072	19
[25]	93.6	-	-
[12]	95	-	15
[26]	95.6	-	-
[27]	95.0	0.238	-
CFFGN (Ours)	96.5	0.104	14

**Table 3:** Ablation study results on the Cornell dataset.

Network	Accuracy (%)		Detection
Architecture	Image-Wise	Object-Wise	Speed (ms)
Baseline	87.56	84.11	30
+DSCM	87.92 (+0.36)	85.23 (+1.12)	11
+CBAM	90.34 (+2.78)	89.65 (+5.54)	36
+MCDM	91.28 (+3.72)	90.54 (+6.43)	33
+BiFPN	92.15 (+4.59)	91.36 (+7.25)	35
CFFGN(Ours)	98.62	96.91	15

**Table 4:** Ablation study results with combined modules on the Cornell dataset.

Network	Accuracy (%)		Detection
Architecture	Image-Wise	Object-Wise	Speed (ms)
Baseline	87.56	84.11	30
+CBAM + MCDM	93.74 (+6.18)	92.59 (+8.48)	39
+CBAM + BiFPN	94.26 (+6.70)	93.15 (+9.04)	41
+MCDN + BiFPN	93.92 (+6.36)	92.87 (+8.76)	38
+CBAM + MCDN + BiFPN	96.16 (+8.6)	95.29 (+11.18)	44
CFFGN(Ours)	98.62	96.91	15

**Table 5:** Robot grasping experiment results.

Object	Grasping Performance	
	Success Rate (%)	Successful Grasps
Thermos	92	23/25
Watch	88	22/25
Stapler	100	25/25
Small express box	100	25/25
Apple	96	24/25
Pencil case	96	24/25
Milk carton	100	25/25
Sunglasses	92	23/25
Wireless mouse	92	23/25
Battery	100	25/25
Average	95.6	23/25

## Data Availability

The data presented in this study are available on request from the corresponding author.

## Abbreviations

The following abbreviations are used in this manuscript:

MCDM	Multi-scale Dilated Convolution Module
BiFPN	Bidirectional Feature Pyramid Network
DSCM	Depth-Separable Convolution Module
CBAM	Convolutional Block Attention Module
CFFGN	Cascaded Feature Fusion Grasping Network
IoU	Intersection over Union
CNN	Convolutional Neural Network
MLP	Multi-Layer Perceptron
ReLU	Rectified Linear Unit
BN	Batch Normalization
